# Clinical characteristics and therapeutic behavior of breast cancer patients using mistletoe therapy consulting a clinic offering integrative oncology: a registry data analysis

**DOI:** 10.1186/s12906-023-04219-x

**Published:** 2023-11-03

**Authors:** Daniel Krüerke, Marianne Schenker, Klazien Matter-Walstra

**Affiliations:** 1 Klinik Arlesheim AG, Pfeffingerweg 1, Arlesheim, CH-4144 Switzerland; 2 Society for Cancer Research, Hiscia Institute, Kirschweg 9, Arlesheim, CH-4144 Switzerland; 3 European Center of Pharmaceutical Medicine, University of Basel, Klingelbergstrasse 61, Basel, CH-4056 Switzerland

**Keywords:** Breast cancer, Complementary medicine, Integrative oncology, Mistletoe, Surgery, Treatment pathway

## Abstract

**Motivation:**

Cancer patients often use complementary and/or alternative medicine, such as mistletoe therapy, alongside conventional cancer therapies. In Switzerland, so far not much is known about treatment patterns of breast cancer patients using integrative oncology. Solid knowledge on complementary care utilization may help to enhance integrative oncology care in Switzerland.

**Methods:**

In this exploratory, descriptive database study, we investigated the treatment pathways of a cohort of breast cancer patients who received mistletoe therapy and were documented in the cancer registry of an anthroposophic Swiss hospital offering integrative oncology treatments.

**Results:**

Patients treated with mistletoe in this cohort are in median 10 years younger than Swiss breast cancer patients as a whole. Only 5.8% of these patients were treated with mistletoe alone, while 60.5% of them supplemented chemotherapy and/or hormone therapy and/or surgery and/or radiation with mistletoe therapy. Nearly 80% of patients started conventional therapy followed by additional mistletoe therapy or started mono mistletoe therapy after completion of conventional therapies. The median time from initial diagnosis to hospital admission (inpatient and/or outpatient) was less than one year. Almost ¾ of the patients were treated in an outpatient setting only.

**Conclusion:**

From our data, it appears that younger breast cancer patients are more likely to use mistletoe therapy simultaneously with or following their conventional medical therapies. The extent to which these patients discuss their mistletoe therapy and eventually other complementary and/or alternative therapies with their primary oncologists is not clear from the data. We therefore recommend that (Swiss) oncologists should openly discuss the desire for integrative oncology therapies, especially with their younger breast cancer patients, in order to find the best holistic care pattern for these patients.

**Supplementary Information:**

The online version contains supplementary material available at 10.1186/s12906-023-04219-x.

## Background

Cancer patients who are open to complementary and/or alternative medicine approaches, and historically this is especially the case in the German-speaking countries of Europe, often use mistletoe therapies (MT) as a complementary and/or alternative treatment [1, 2]. The aim of mistletoe treatment may be to reduce cancer related fatigue, improve quality of life, prolong overall survival or to ease side effects of conventional medicine (COM) treatments [3, 4]. Although the effectiveness of MT in cancer treatments is still controversially discussed [5–10], their use in clinical practice is a reality [11]. MT is mostly used as a complementary treatment alongside or following COM treatments. To this purpose, the patient either organizes the complementary treatments him/herself or is within an integrative oncology (IO) setting, where a combination of complementary medicine and conventional treatments is professionally coordinated with the patient, as comprehensively defined in a recent article [12]. For example, a Swiss study with breast cancer survivors found that 16% of the patients used mistletoe therapies [13], and in a Swiss study of end-of-life care for cancer patients, nearly 12% had used mistletoe [14]. Cancer patients are often reluctant to discuss their complementary treatments with their primary treating oncologist [15, 16]. Surveys have also shown that conventionally treating physicians greatly underestimate their patients’ use of complementary therapies [17]. The use of complementary therapies also increases as patients become increasingly dissatisfied with the side effects of conventional treatments [18]. Furthermore, clinical trials that are COM-only studies occasionally and unknowingly include patients using complementary treatments, which can lead to questionable results and interpretations.

To what extent treating oncologists of conventional therapies know about the use of mistletoe in their cancer patients (if the practitioner does not practice integrative oncology) or discuss this possibility is unknown.

The sometimes highly emotional controversies between proponents and opponents of complementary and/or alternative medicine (CAM) and COM among health professionals, but also journalists, politicians, and legislators, create irritation among patients and integrative treating physicians [19].

The Clinic Arlesheim is a Swiss anthroposophic hospital offering IO treatments for cancer patients, including drug therapies, of which MT is the most used complementary anti-cancer treatment. In addition, complementary drug and/or non-drug therapies are offered that focus on the physical and psychological dimensions of the cancer patients. These complementary treatment options can be used by the patients according to their own preference [20, 21]. Overall, a multimodal integrative treatment concept usually results from conventional and individually adapted complementary treatments. The latter are therefore very diverse and, especially in the outpatient setting, very unreliably documented in patient records. The hospital operates its own, cancer registry of the Clinic Arlesheim (CRCA), which is based on the information in the patient records [22]. This registry uses the documentation system QuaDoSta (quality assurance, documentation, and statistics), a flexible, platform-independent, open-source database for oncological documentation [23, 24]. The CRCA offers a good opportunity to investigate care patterns of cancer patients who seek for complementary and/or alternative treatments, especially MT for their cancer. Since virtually all cancer patients at Clinic Arlesheim are treated with MT, this therapy is also the most completely and extensively documented.

In Switzerland, not much is known so far about care patterns of breast cancer patients using IO. Therefore, we evaluated real-world data documented in the CRCA on clinical and therapeutic characteristics from included breast cancer patients which use MT as a complementary therapy. The aim of this evaluation is to obtain an improved understanding of the treatment behavior of breast cancer patients seeking complementary treatments. Results of this work should help conventional health care professionals in Switzerland to become more aware of these facts and encourage them to support an integrative care system for Swiss cancer patients, in general.

## Methods

This study is an exploratory and descriptive analysis of existing non-genetic health-related routine clinical data of a cohort with breast cancer patients.

For our analyses, we considered all female in- and outpatients of the Clinic Arlesheim whose disease trajectories were documented in the registry (CRCA) with the following inclusion and exclusion criterions.

inclusion criteria:

- Breast cancer diagnosis: (ICD10/C50)
- Females.
- First Diagnosed between 2008/01/01-2017/10/01.
- Valid written informed consent to the further use of their non-genetic health data.
- Outpatient or inpatient treatment in the first year after registration at the Clinic Arlesheim.

Exclusion criteria:

- Only medical consultation within the first year after registration at the Clinic Arlesheim.
- Only telephone contact or e-mail contact within the first year after registration at the Clinic Arlesheim.
- No mistletoe treatment.
- No malignant tumor.

The evaluations cover the periods from the initial diagnosis to the date of death or until the database extract on 2019/10/01. The data include information on demographic, the clinical status of the patients as well as on disease progression, including complementary and COM treatments within the clinic, but also on treatments being performed outside of the clinic. The database also contains detailed information on MT, as the most important complementary cancer treatment.

In the first step, the CRCA database entries were checked for completeness and plausibility. Based on patient records in paper form or electronic medical records from the hospital information system, implausible data were reviewed and corrected accordingly, missing data were completed according to the patients’ dossier. The following variables were included for the evaluation: age at initial diagnosis, hormone and HER2 status, start date and type of applied therapies including chemotherapy and/or hormone-therapy (CHT), surgery (Surg), radiotherapy (RT), and MT, start of outpatient or inpatient care in the hospital Clinic Arlesheim, as well as survival status. With the use of these variables therapy combinations, treatment pathways and time from initial diagnosis until start of treatment in the clinic were established.

As this study did not compare the included cohort with patients not using IO the evaluation of the data was explicitly performed in a descriptive manner. Kaplan Meyer curves were created to describe survival in the data available. These evaluations have neither the claim nor the basis to provide evidence-based indications of better or worse treatment methods. We would like to point out that the situations depicted do not allow any generalizations to be made in this regard. The evaluation was performed in SAS9.4®.

## Results

We identified a cohort of 1031 breast cancer patients in the CRCA who met the inclusion criteria. This cohort was evaluated until database extraction on 2019/10/01. The mean age of patients at initial diagnosis was 55.40 (STDEV 11.47, median 54.7) and most patients had HR+/HER2- hormone status (54.6%, see Table 1). The median age of patients in the cohort at initial diagnosis is almost 10 years younger (54.7) than that of the overall Swiss breast cancer population (64.2) [25]. The distribution of the hormonal status differs somewhat from reports of the United States [26]. In particular, the proportion of patients with HR+/HER2- status is lower in our study cohort than in the U.S. study (54.6% versus 72.7%), whereas the group of HR+/HER2 + patients is larger in our study cohort (18.9% versus 10.3% in the U.S. study).

**Fig. 1:**
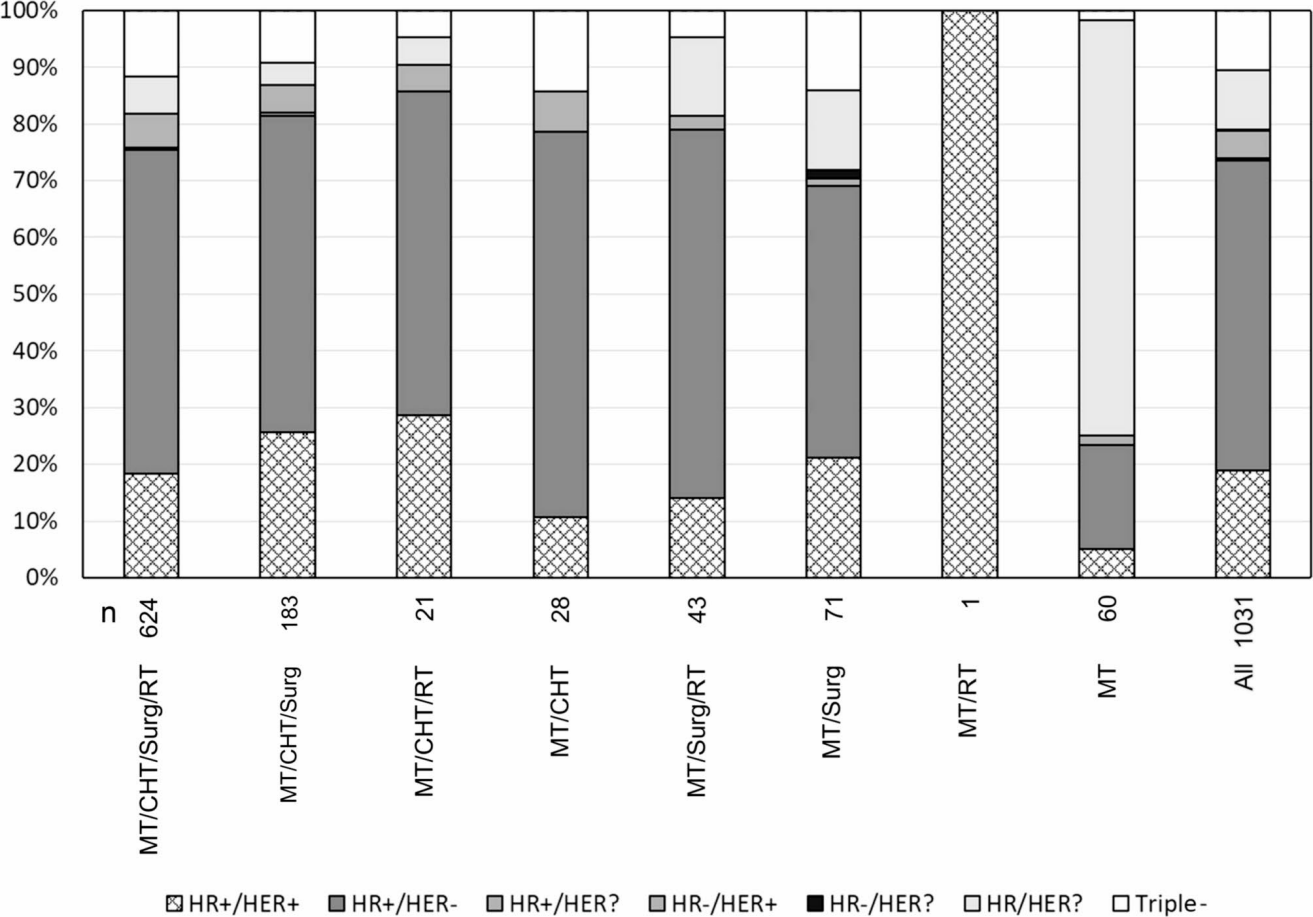
Combined clinical and therapeutic characteristics Legend: MT = mistletoe therapy, CHT = chemotherapy and/ or hormone therapy. Surg = surgery, RT = radiotherapy, HR = estrogen and/or progesterone receptor, HER = HER2 receptor, Triple-=estrogen, progesterone and HER2 receptor negative

The majority of the patients (60.5%) were treated with mistletoe therapy, chemotherapy and/or hormone therapy, surgery and radiotherapy (MT/CHT/Surg/RT, see Table 1). Only 5.8% patients were treated with MT alone. This group is a combination of patients who explicitly rejected therapies other than MT, or who started MT and later received any conventional therapy recommended by the clinic but not recorded in the database or patient documentation. The distribution of the HR/HER2 types over the therapy combinations (Fig. 1) largely corresponds to the general HR/HER2 distribution. None of the HR/HER2 types was particularly overrepresented in any of the therapy combinations. In the group of patients only known to have received mistletoe (n = 60), the HR/HER2 type was mostly unknown.

**Table 1:** Cohort description for cancer type and therapy

	Age at Diagnosis				
	n	%	mean (years)	STDEV	Median
All	1031		55.4	11.4	54.7
HR+/HER2+	195	18.9	55.8	11.2	55.6
HR+/HER2-	563	54.6	56.0	11.4	55.1
HR+/HER2?	4	0.4	50.1	8.4	52.5
HR-/HER2+	51	4.9	52.1	11.0	50.5
HR-/HER2?	1	0.1	53.2	.	53.2
HR/HER2?	109	10.6	55.4	11.5	53.9
Triple-	108	10.5	53.5	11.7	53.0
**Therapy combinations**					
MT/CHT/Surg/RT	624	60.5	55.0	10.81	54.5
MT/CHT/Surg	183	17.7	55.1	12.15	53.1
MT/CHT/RT	21	2.0	54.4	10.10	53.9
MT/CHT	28	2.7	58.0	14.24	59.3
MT/Surg/RT	43	4.2	58.4	11.96	58.7
MT/Surg	71	6.9	57.7	13.08	55.9
MT/RT	1	0.1	80.0	.	80.0
MT	60	5.8	55.1	11.26	53.3

Legend: HR = estrogen and/or progesterone receptor, HER = Her2 receptor, Triple-=estrogen, progesterone and Her2 receptor negative. MT = mistletoe therapy, CHT = chemotherapy and/or hormone therapy, Surg = surgery, RT = radiotherapy. For details, see Fig. 1 “Combined clinical and therapeutic characteristics”

Most of the patients were treated as outpatients (72.2%, see Table 2) and 79.1% of the patients initially started with a conventional therapy (CHT, surgery, or radiotherapy) and complemented it with MT in a later phase of the treatment pathway or started MT after completion of conventional therapies (see Tables 2 and 3). The most common treatment pathway (n = 333) was surgery followed by a combination of CHT (with or without radiotherapy) and MT. The second common treatment pathway includes those patients who started MT after completion of all conventional therapies (n = 305).

**Table 2:** Type of care and start of MT or COM by hormone and Her2 status

		Total	HR+/ HER2+	HR+/ HER2-	HR+/ HER2?	HR-/ HER2+	HR-/ HER2?	HR?/ HER2?	Triple-
Outpatient	n	744	146	403	3	38	1	82	71
	%	72.2	19.6	54.2	0.4	5.1	0.1	11.0	9.5
Outpatient and inpatient	n	239	42	138	1	11	0	16	31
	%	23.2	17.6	57.7	0.4	4.6	.	6.7	13.0
Inpatient	n	35	7	17	0	2	0	3	6
	%	3.4	20.0	48.6	.	5.7	.	8.6	17.1
No data on type of care	n	13	0	5	0	0	0	8	0
	%	1.3	.	38.5	.	.	.	61.5	.
Start with MT	n	202	33	92	0	13	1	46	17
	%	19.6	16.3	45.5	.	6.4	0.5	22.8	8.4
Start with COM	n	816	161	470	4	38	0	52	91
	%	79.1	19.7	57.6	0.5	4.7	.	6.4	11.2
No data on start of therapy	n	13	1	1	0	0	0	11	0
	%	1.3	7.7	7.7	.	.	.	84.6	.

Outpatient and inpatient treatments and start of COM or MT treatments in the hospital Clinic Arlesheim by hormone and Her2 status. Legend: MT = mistletoe therapy, COM = chemotherapy and/or hormone therapy and/or surgery and/or radiotherapy, HR = estrogen and/or progesterone receptor, HER = Her2 receptor, Triple-=estrogen, progesterone and Her2 receptor negative, Start with MT=patients started with MT with or without a combination of any type of COM, Start with COM = patients started with any type of COM and complemented or continued with MT later in the course of disease, see Table 3.

**Table 3:** Care pathway

Patients start immediately with mistletoe therapy	n = 202	19.6%
(MT,CHRT)	14	7%
(MT,CHRT) > Surg	23	11%
(MT,CHRT,Surg)	50	25%
(MT,Surg)	9	4%
(MT,Surg) > CHRT	23	11%
MT	50	25%
MT>(CHRT,Surg)	2	1%
MT > CHRT	3	1%
MT > CHRT > Surg	3	1%
MT > Surg	10	5%
MT > Surg > CHRT	15	7%
**Patients start with any type of conventional therapy**	**n = 816**	**79.1%**
(CHRT,Surg) > MT	15	1.8%
CHRT>(MT,Surg)	9	1.1%
CHRT > MT	24	2.9%
CHRT > MT > Surg	5	0.6%
CHRT > Surg > MT	39	4.8%
Surg>(MT,CHRT)	333	40.8%
Surg > MT	59	7.2%
Surg > MT > CHRT	27	3.3%
Surg > CHRT > MT	305	37.4%
No data^1)^	13	1%

Legend MT = Mistletoe therapy, CHRT = Chemotherapy and/or hormone therapy and/or radiotherapy, Surg = surgery, ( ) = combined therapy, “>”=proceeding therapy, ^1)^it is known that these patients received at least one mistletoe treatment, but not at what time in their treatment pathway.

**Fig. 2:**
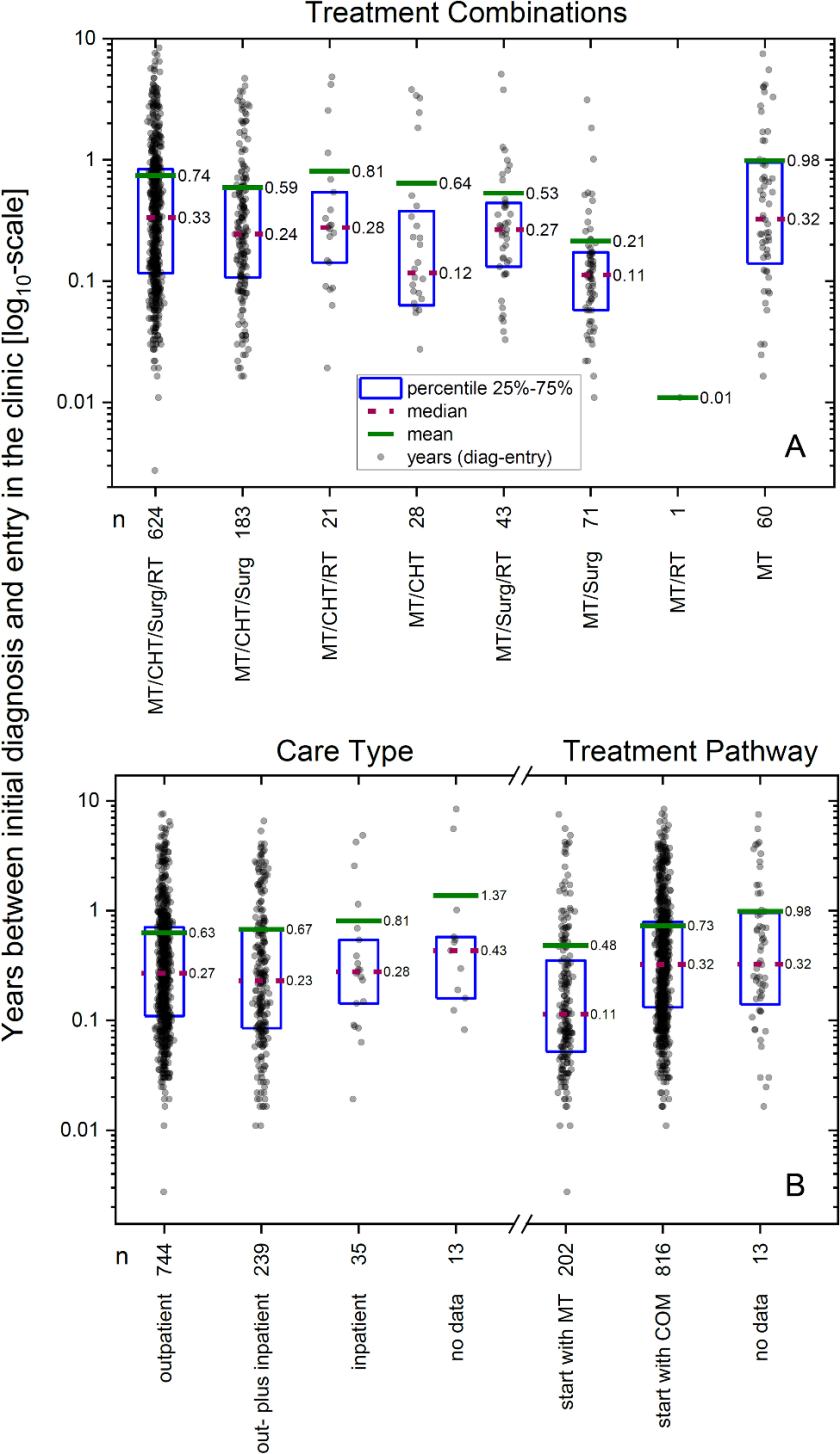
Time from initial diagnosis to start of treatment in the Clinic Duration between diagnosis and admission in years for: (**A**) Different treatment combinations. Legend: (**A**) MT = mistletoe therapy, CHT = chemotherapy and/or hormone therapy, Surg = surgery, RT = radiotherapy. (**B**) Type of care and treatment pathways. Legend: COM = chemotherapy and/or hormone therapy and/or surgery and/or radiotherapy, start with MT=patients started with MT with or without a combination of any type of COM therapies, start with COM = patients started with any type of COM therapy and complemented or continued with MT later in the course of disease, see Table 3. Note that logarithmic scaling was used for the time interval. If linear scaling were used, the values for the distribution characteristics would be indistinguishable due to skewed distributions toward short time spans

The mean time from initial diagnosis to hospital admission (in-/or outpatient) is 0.68 years (STDEV 1.08, median 0.27, see also supplementary material Table 1). This time span is particularly long for patients known to have received MT only (n = 60) or for those who were treated as inpatients only (compare Fig. 2). Furthermore, the distribution of time from diagnosis to hospital admission is strongly skewed, with a few patients with a very long time-interval driving up the mean values.

**Fig. 3:**
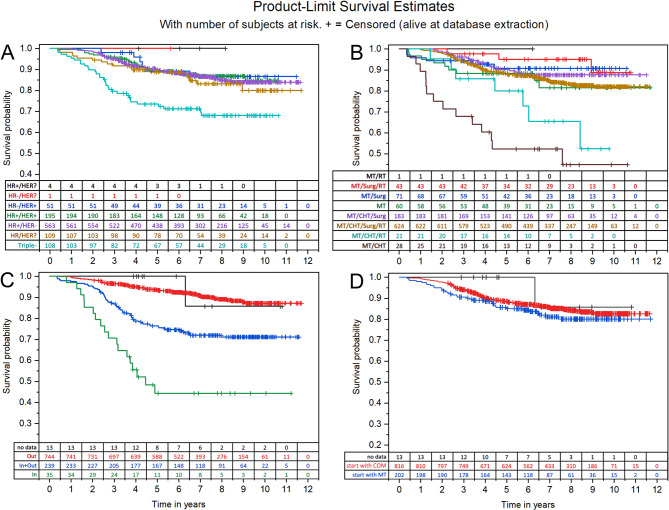
Survival curves according to A: HR/HER type, B: therapy combination, C: care type, D: treatment pathway Legend: (**A**) HR = estrogen and/or progesterone receptor; HER = HER2 receptor; Triple-=estrogen, progesterone and Her2 receptor negative. (**B**) MT = mistletoe therapy; CHT = chemotherapy and/ or hormone therapy; Surg = surgery; RT = radiotherapy. (**C**) Out = outpatient; In = inpatient; In + Out = inpatient and outpatient; no data = unknown. (**D**) COM = chemotherapy and/or hormone therapy and/or surgery and/or radiotherapy, start with MT = patients started with MT with or without any type of COM therapies; start with COM = patients started with any type of COM therapy and complemented or continued with MT later in the course of disease, see Table 3

Regarding survival (compare Fig. 3), Triple negative patients (A), patients treated with CHT and MT without surgery and without radiotherapy (B), as well as patients treated as ambulatory and stationary or stationary only (C) showed worse survival probabilities. Whether patients started MT immediately or later in the course of treatment did not seem to influence the probability of survival (D).

## Discussion

In this paper, we describe a unique evaluation of the treatment course of breast cancer patients seeking an IO treatment with mistletoe in the anthroposophic hospital Clinic Arlesheim in Switzerland. Due to the detailed documentation of patient characteristics and clinical treatments in the register for IO [22–24], we were able to describe the care pattern of these patients. The first distinctive characteristic of the treated patients is that they are in median almost 10 years younger at initial diagnosis than the general breast cancer population in Switzerland [25]. This is consistent with the results of a systematic review by Wanchai et al. [27], who reported that breast cancer patients using CAM therapies were likely to be younger.

A recent study also confirmed that breast cancer patients using alternative oncology treatments tended to be younger and that more than half of them had not discussed the use of alternative therapies with their primary oncologists. This can lead to significant problems with drug interactions, especially with chemotherapy [28].

The distribution of HR/HER2 status in our cohort differs somewhat from data for the United States [26]. Data on this distribution are not available for Switzerland. Therefore, it is unclear whether the distribution found is a Swiss characteristic or is due to certain HR/HER2 populations seeking proportionally less or more mistletoe therapy. A recent study suggests that breast cancer appears to be more frequently triple negative in younger women, which somewhat contradicts our results. Our cohort was in general younger but had a lower triple negative proportion as reported by Shah et al. [29].

Treatment combinations for conventional treatments likely depend on the patient’s breast cancer type, and almost all patients (94.2%) received one or more different conventional therapies (chemo- and/or hormone therapy, surgery, radiotherapy). However, whether any of the conventional treatments (or combinations of treatments) are over or under-represented in this cohort compared to patients not using MT is unknown, as such data or not available for Switzerland. Very few patients in the cohort have rejected conventional therapies altogether.

Analysis of treatment courses showed that nearly 80% of the patients started with conventional therapy and added mistletoe to further conventional therapies at a later phase of treatments or (second most often) started with MT after completion of conventional therapies. Whether patients started MT immediately in addition to conventional therapy or later in the course of treatment did not seem to have an influence on the survival probability. However, their treating IO physician should advise those patients who start with MT only, potentially delaying COM treatment, that this may adversely affect their survival.

This pattern of care and the survival analyses somewhat contradict the impression expressed by various physicians in antroposophic or alternative medicine clinics that cancer patients often seek out CAM when they become treatment resistant and are in a terminal state. To investigate this hypothesis, complete TNM information of patients would have been helpful, but this information was sparsely documented in the CRCA, which is a known problem for many cancer registries [30, 31]. This probably biased impression is even further invalidated by the fact that most patients are treated only as outpatients (72.2%) and, on average, come to the clinic within one year of diagnosis. On the other hand, the group of patients treated exclusively as inpatients (n = 35) might contain more treatment-resistant patients, as the time between diagnosis and hospitalization for these patients was on average 1.6 years and their survival probability was worse than for patients treated as outpatients.

It is problematic that cancer patients often not discuss their complementary therapies with their primary oncologists. In addition, both positive and negative interactions between mistletoe therapy and conventional cancer therapies have been reported from in vitro studies [32, 33]. However, clinical studies that clearly demonstrate such interactions between MT and chemotherapies or hormone therapies in the treatment of patients are still pending. Mansky already pointed this out in 2002, which has not changed until now [34]. We therefore recommend that (Swiss) oncologists familiarize themselves with alternative treatment methods and appropriate IO counselling services, in order to be able to openly and competently discuss the desire for alternative therapies, especially with younger breast cancer patients [17]. On the other hand, IO physicians should point out potential interactions and encourage their patients to mention their IO treatments when talking with their COM oncologists as well. Improved two-sided communication in this regard will build trust and help identify the best holistic treatment patterns for these patients.

This analysis of breast cancer patient data has several limitations. First, the data from the register had to be evaluated in several iterations to be corrected and completed with data from the patient dossiers. With a cohort of 1031 patients, this was manageable, but the extent to which the existing data in the register where correct can only be guessed at since only implausible or missing data were corrected or completed. Our intensive investigations for plausibility and completeness of data confirmed a general weakness of cancer registries to frequently contain incorrect or incomplete data [30, 35]. Second, we could not compare some of our results with a breast cancer population not using mistletoe because the general Swiss cancer registries do not provide sufficiently detailed information on treatment strategies and patterns of care.

## Conclusions

This evaluation of real-world data on the clinical and therapeutic characteristics of breast cancer patients using IO confirms that they are younger than the overall breast cancer population in Switzerland. In addition, only very few patients relied on MT alone, so the use of MT is a complementing rather than an “alternative” therapy. The analysis of the registry data revealed however a general problem with database studies. Very often, what would have been good to document for evaluating specific issues is determined only afterwards. When using clinical registry data to answer scientific questions, combining them with health insurance data could be a useful extension. Future research should address how communication and consultation with COM and IO physicians occurs among cancer patients in general and how it occurs when the COM and IO physicians providing care are already working well together.

### Electronic supplementary material

Below is the link to the electronic supplementary material.

Supplementary Material 1

## Data Availability

The data sets analyzed in the current study are not publicly accessible due to data protection regulations. The data originate from the cancer registry of the hospital Clinic Arlesheim. They were provided by the patients with their written consent for further use for research purposes. They can be analyzed in this context, and the results can be published, but not the clinical raw data themselves. However, the pseudonymized dataset used for the analysis in this paper can be made available upon justified request by the corresponding author with the permission of the hospital management and the data protection officer of the hospital Clinic Arlesheim.

## Declarations

### Clinic Arlesheim

The “Klinik Arlesheim” is an anthroposophical acute hospital in north-western Switzerland. It practices an anthroposophical-medical extension of conventional medicine in diagnosis, treatment and therapy in a comprehensive inpatient and outpatient care program. In addition to conventional drug treatments, which may include chemotherapies, various anthroposophic drug therapies, e.g. with mistletoe preparations, and various non-drug therapies are often used in combination. From an anthroposophical point of view, holistic treatments on a physical, psychological and spiritual level are targeted.

## Abbreviations

C50: Malignant neoplasm of breast (ICD10 classification code, see below)
CAM: Complementary and alternative medicine
CHT: Chemotherapy and/or hormone therapy
CHRT: Chemotherapy and/or hormone therapy and/or radiotherapy
COM: Conventional medicine. In this work, COM treatment refers to any combination of radiotherapy, chemotherapy and/or hormone therapy, and surgery
CRCA: Cancer registry of the Clinic Arlesheim
ICD10: International Statistical Classification of Diseases and Related Health Problems list by the World Health Organization
IO: Integrative oncology
HER2: Human epidermal growth factor receptor 2
HR: Hormone receptor oestrogen and/or progesterone
MT: Mistletoe therapy
QuaDoSta: Database system:quality assurance, documentation, and statistics
RT: Radiotherapy
STDEV: Standard deviation
Start with COM: Patients first start with any type of conventional therapy and complement or proceed with mistletoe later in the course of disease
Start with MT: Patients immediately start with MT with or without a combination of any type of conventional therapy
Surg: Surgery
