# Supplementary material for: Clinical characteristics and therapeutic behavior of breast cancer patients using mistletoe therapy consulting a clinic offering integrative oncology: a registry data analysis
Source: BMC Complement Med Ther. 2023 Nov 3;23:395. doi: 10.1186/s12906-023-04219-x (PMC10623842; doi:10.1186/s12906-023-04219-x)
Supplement: Supplementary file 1 — Supplementary Material 1 [file 12906_2023_4219_MOESM1_ESM.docx]

**Additional material**

Tabel 1 Time from initial diagnosis to start of treatment in the Clinic in years.

|  | N | Mean in  years | Median |  |  | Max | Min | SDEV |
| --- | --- | --- | --- | --- | --- | --- | --- | --- |
|  |  |  |  | 25% - 75% Percentile | |  |  |  |
| All patients with CHT | 856 | 0.71 | 0.29 | 0.11 | 0.77 | 8.40 | 0.00 | 1.08 |
| All patients without CHT | 175 | 0.55 | 0.17 | 0.08 | 0.45 | 7.50 | 0.00 | 1.08 |
| All patients with Surg | 921 | 0.66 | 0.27 | 0.11 | 0.72 | 8.40 | 0.00 | 1.04 |
| All patients without Surg | 110 | 0.85 | 0.25 | 0.09 | 0.70 | 7.50 | 0.00 | 1.39 |
| All patients with RT | 688 | 0.73 | 0.32 | 0.12 | 0.80 | 8.40 | 0.00 | 1.12 |
| All patients without RT | 343 | 0.58 | 0.19 | 0.08 | 0.54 | 7.50 | 0.00 | 1.00 |
| MT/CHT/Surg/RT | 624 | 0.74 | 0.33 | 0.12 | 0.84 | 8.40 | 0.00 | 1.12 |
| MT/CHT/Surg | 183 | 0.59 | 0.24 | 0.11 | 0.59 | 4.70 | 0.00 | 0.87 |
| MT/CHT//RT | 21 | 0.81 | 0.28 | 0.14 | 0.54 | 4.84 | 0.02 | 1.35 |
| MT/CHT | 28 | 0.64 | 0.12 | 0.06 | 0.36 | 3.79 | 0.00 | 1.14 |
| MT/Surg/RT | 43 | 0.53 | 0.27 | 0.14 | 0.43 | 5.09 | 0.03 | 0.93 |
| MT/Surg | 71 | 0.21 | 0.11 | 0.06 | 0.17 | 3.12 | 0.00 | 0.43 |
| MT//RT | 1 | 0.01 | 0.01 | . | . | 0.01 | 0.01 | . |
| MT | 60 | 0.98 | 0.32 | 0.15 | 0.94 | 7.50 | 0.00 | 1.51 |
| Outpatient | 744 | 0.63 | 0.27 | 0.11 | 0.70 | 7.59 | 0.00 | 0.99 |
| Outpatient and inpatient | 239 | 0.67 | 0.23 | 0.08 | 0.69 | 6.55 | 0.00 | 1.03 |
| Inpatient | 35 | 1.63 | 0.72 | 0.15 | 2.95 | 7.33 | 0.00 | 1.82 |
| no data on type of care | 13 | 1.37 | 0.43 | 0.16 | 0.58 | 8.40 | 0.00 | 2.56 |
| Start with MT | 202 | 0.49 | 0.11 | 0.05 | 0.36 | 7.50 | 0.00 | 1.04 |
| Start with COM | 816 | 0.72 | 0.32 | 0.13 | 0.78 | 8.40 | 0.00 | 1.08 |
| No data on start of therapy | 13 | 1.05 | 0.65 | 0.16 | 1.17 | 5.54 | 0.00 | 1.54 |
| HR+/HER+ | 195 | 0.66 | 0.33 | 0.12 | 0.77 | 5.30 | 0.00 | 0.93 |
| HR+/HER- | 563 | 0.63 | 0.23 | 0.10 | 0.65 | 8.40 | 0.00 | 1.06 |
| HR+/HER? | 4 | 0.19 | 0.19 | 0.07 | 0.31 | 0.33 | 0.03 | 0.16 |
| HR-/HER+ | 51 | 0.65 | 0.30 | 0.09 | 0.65 | 3.17 | 0.00 | 0.84 |
| HR-/HER? | 1 | 0.07 | 0.07 | . | . | 0.07 | 0.07 | . |
| HR/HER? | 109 | 0.97 | 0.35 | 0.13 | 0.92 | 7.59 | 0.00 | 1.48 |
| Triple- | 108 | 0.74 | 0.28 | 0.11 | 0.85 | 5.66 | 0.02 | 1.04 |

Legend: HR =estrogen and/or progesterone receptor, HER=HER2 receptor, Triple-=estrogen, progesterone and Her2 receptor negative, MT=mistletoe therapy, CHT=chemotherapy and/ or hormone therapy, Surg=surgery, RT=radiotherapy, COM=chemotherapy and/or hormone therapy and/or surgery and/or radiotherapy,, Start with MT=patients start with MT with or without any type. of COM, Start with COM=patients start with any type of COM and complemented or continued with MT later in the course of disease.
